# Sodium-glucose co-transporter 2 inhibitor add-on therapy for metformin delays diabetic retinopathy progression in diabetes patients: a population-based cohort study

**DOI:** 10.1038/s41598-023-43893-2

**Published:** 2023-10-10

**Authors:** Jing-Xing Li, Yu-Tung Hung, Henry Bair, Shu-Bai Hsu, Chung-Yi Hsu, Chun-Ju Lin

**Affiliations:** 1 Department of Internal Medicine, Taipei Veteran General Hospital, Taipei, Taiwan; 2 School of Medicine, China Medical University, Taichung, Taiwan; 3 Graduate Institute of Clinical Laboratory Sciences and Medical Biotechnology, National Taiwan University, Taipei, Taiwan; 4 Institute of Public Health, China Medical University Hospital, Taichung, Taiwan; 5 Department of Ophthalmology, China Medical University Hospital, Taichung, Taiwan; 6 Byers Eye Institute, Stanford University School of Medicine, Stanford, CA USA; 7 Department of Nursing, China Medical University Hospital, Taichung, Taiwan; 8 School of Nursing, China Medical University, Taichung, Taiwan; 9 Graduate Institute of Biomedical Sciences, China Medical University, Taichung, Taiwan; 10 Department of Optometry, Asia University, Taichung, Taiwan

**Keywords:** Endocrinology, Epidemiology

## Abstract

To investigate how sodium-glucose co-transporter 2 inhibitors (SGLT2is) add-on therapy for metformin affects diabetic retinopathy (DR) progression in patients with type 2 diabetes mellitus (T2DM). This nationwide population-based study conducted from January 1, 2016, to December 31, 2018 involved 3,432,911 adults with T2DM in Taiwan. To adjust for potential confounders, data on sex, age, income, comorbidities, diabetes complication severity index score, staging of kidney disease, anti-diabetic medications, and index year were included. The outcome was DR progression, determined by procedure codes or the addition of ICD-9-CM or ICD-10-CM codes to the medical records of the patients during the study. Sensitivity analyses were performed to validate the findings. The adjusted hazard ratio (aHR) of DR progression was 0.89 for the SGLT2is add-on group, relative to the control group [95% confidence interval (CI) 0.81–0.99, *P* = 0.026]. The Kaplan–Meier curve of the cumulative incidence rate showed that the cumulative incidence of DR progression was considerably decreased in the SGLT2is cohort (log-rank *P* = 0.0261). The use of SGLT2is for less than 1 year and 1–2 years were associated with a significant increase in the risk of DR progression (aHR 1.56 and 1.88, respectively); however, the risk markedly reduced if the SGLT2is regimen was used for more than 2 years (aHR 0.41, 95% Cl 0.35–0.48; *P* < 0.001). The serial sensitivity analysis showed consistent findings. The aHR of DR progression was 0.82 for the SGLT2is cohort relative to the non-SGLT2is cohort based on the fundoscopy or indirect ophthalmoscopy findings within 1 year before the outcome date (95% Cl 0.71–0.95; *P* = 0.009). Co-administration of metformin and SGLT2is may reduce the risk of DR progression. Short-term use of SGLT2is may markedly increase the risk of DR, whereas prolonged use SGLT2is may significantly decrease it.

## Introduction

Diabetic retinopathy (DR) is the most common complication of diabetes mellitus (DM) and the leading cause of blindness. Among individuals with diabetes, the global prevalence of DR was 22.27%. In 2020, the number of adults with DR was estimated to be 103.12 million; by 2045, the number is projected to increase to 160.50 million^1^. The pathophysiology of DR is complex, and its underlying mechanism is not yet fully understood. The currently proposed pathogenic mechanisms include chronic inflammation, retinal hemodynamic alterations, oxidative stress, gene polymorphisms, and neurodegenerative changes^2^.

Several anti-diabetic medications (ADMs) have been developed, including biguanide (metformin), sulfonylureas (SUs), thiazolidinediones (TZDs), glucagon-like peptide 1 receptor agonists (GLP-1RAs), dipeptidyl peptidase-4 inhibitors (DPP4is), and subcutaneous/intravenous insulin^3^. Sodium-glucose co-transporter-2 inhibitors (SGLT2is), such as empagliflozin, dapagliflozin, and canagliflozin, are currently widely available and clinically used for the treatment of patients with type 2 diabetes mellitus (T2DM)^4^. The mechanism of action of SGLT2is is the reversible blockage of SGLT2 in the proximal collecting duct, which, in turn, inhibits glucose reabsorption and facilitates its excretion in urine^5^. In current clinical practice, SGLT2is are mostly used to regulate blood glucose in individuals with T2DM who are prone to congestive heart failure because of their associated beneficial cardiovascular outcomes^6^.

Non-insulin anti-hyperglycemic agents have been generally found to have moderate or no beneficial effects on DR in T2DM patients^7^; among them, evidence of the effects of SGLT2is on DR is limited. In an experimental study, ipragliflozin delayed the progression of retinopathy in spontaneous diabetic Torii fatty rats^8^. A case report revealed that diabetic macular edema (DME) decreased significantly after 16 weeks of treatment with ipragliflozin^9^. SGLT2is may also be associated with a lower risk of DR than DPP4is^10^. However, there have been no reports of a large-scale population study on the effects of SGLT2is on DR, to the best of our knowledge, and real-world outcomes have not been validated. Accordingly, this study aimed to assess the risk of DR in patients treated with SGLT2is as add-on therapy.

## Methods

### Study population

More than 99% of the Taiwanese population joined the National Health Insurance (NHI) plan as a mandatory insurance system. The NHI Research Database (NHIRD) contains data about the insured, including age, sex, place of residence, income, diagnosis, medications, and medical procedures. Several studies have used this database over the past two decades^11–13^. The disease codes in this database were based on the International Classification of Diseases, Ninth and Tenth Revision, Clinical Modification (ICD-9-CM and ICD-10-CM) for outpatient and inpatient settings. The present study adhered to the principles of the Declaration of Helsinki and followed the Strengthening the Reporting of Observational Studies in Epidemiology (STROBE) guidelines for reporting observational studies^14^. The identifiable information of caregivers and patients was scrambled and encrypted before data retrieval. Since only se-identified information was obtained, informed consent was waived by the institutional review board of China Medical University Hospital. This study protocol was approved by the institutional review board of the China Medical University Hospital research ethics committee (ID number CMUH110-REC3-133).

### Study design

We identified patients diagnosed with T2DM within January 1, 2016 and December 31, 2018 as the study participants of this retrospective cohort study, and the follow-up period is from January 1, 2016 to December 31, 2019. The diagnosis of T2DM was based on ICD codes (ICD-9-CM codes 250.x0, 250.x2; ICD-10-CM code E11) for at least two outpatient visits or one hospitalization record. Participants were eligible for this study if they met the following inclusion criteria: (1) age of 40 or more years and (2) diagnosis of T2DM and metformin monotherapy for at least 90 cumulative days before 2016. The exclusion criteria were as follows: (1) age less than 40 years or absence of information on sex or age; (2) history of DR according to any of the procedure codes or ICD-9-CM and ICD-10-CM codes mentioned below (Main Outcomes section); (3) use of an SGLT2is for less than 90 cumulative days; (4) use of insulin during the study period; and (5) follow-up period less than 1 year. The case group included patients treated with SGLT2is more than 90 cumulative days. The control group was selected from patients with T2DM during the same period who were treated with metformin but not treated with SGLT2is. We set the index date for the SGLT2is users as the first date of prescription with SGLT2is, and the index date was assigned to the matched non-SGLT2is individuals with the same enrollment criteria. The use of any other oral ADMs during the study period, such as SUs, TZDs, GLP-1RAs, or DPP4is were included. The study period ranged from January 1, 2016 to December 31, 2018, and once SGLT2i was use, the period of SGLT2i was tracked to December 31, 2019, death or withdrawal from NHIRD.

### Main outcomes

We compared the incidence of DR among the cases (SGLT2is cohort) and controls (non-SGLT2is cohort) during the follow-up. The outcomes of DR progression were defined as any of the following: (1) panretinal photocoagulation (60003C and 60004C), macular photocoagulation (60001C and 60002C), intravitreal injection (86201C), pars plana vitrectomy (86206C, 86207 B, 86414 B, and 86415 B), or photocoagulation combined with pars plana vitrectomy (86409 B, 86410 B); or (2) addition of any of the ICD-9-CM and ICD-10-CM codes of the following to the medical record during the study period: vitreous hemorrhage (ICD-9-CM code 379.23; ICD-10-CM code H43.1), retinal detachment (ICD-9-CM 361.0, 361.81; ICD-10-CM H33.0, H33.4), macular edema (ICD-9-CM code 362.83; ICD-10-CM codes E11.3x1, H35.81), and neovascular glaucoma (ICD-9-CM codes 365.41, 365.42, 365.43, 365.51, 365.63, 365.89, 365.59, 365.60, 365.61, 365.64; ICD-10-CM codes H40.5, H40.89). The cumulative incidence of DR of the patients treated with SGLT2is and those who were not.

### Potential confounding factors

Potential confounders that may affect the development of DR were identified. The data of the SGLT2is and non-SGLT2is cohorts assessed included sex; age; income; diabetes complication severity index (DCSI) score; comorbidities; anti-diabetic drugs including SUs, TZDs, GLP-1RAs, and DPP4is; types of ADMs; and index year. The comorbidities in this study were hypertension (ICD-9-CM codes 401–405; ICD-10-CM codes I10–I13, I15, N26.2), dyslipidemia (ICD-9-CM code 272; ICD-10-CM codes E71.30, E75.21, E75.22, E75.24, E75.3, E75.5, E75.6, E77, E78.0, E78.1, E78.2, E78.3, E78.4, E78.5, E78.6, E78.70, E78.79, E78.8, E78.9), coronary artery disease (CAD) (ICD-9-CM codes 410–414; ICD-10-CM codes I20–I25), cerebrovascular accident (ICD-9-CM codes 430–438; ICD-10-CM codes I60–I69), liver cirrhosis (ICD-9-CM code 571.5; ICD-10-CM codes K74.0, K74.1, K74.2, K74.6), chronic kidney disease (ICD-9-CM code 585; ICD-10-CM code N18), and obesity (ICD-9-CM codes 278, 783.1; ICD-10-CM codes E66.09, E66.1, E66.8, E66.9, E66.01, E66.2, E65, E67.0, E67.1, E67.3, E67.2, E67.8, E68, R63.5). Current cigarette smoking was also included (ICD-9-CM codes 305.1, V15.82; ICD-10-CM codes F17.2, Z87.891). Higher scores indicate a greater risk of mortality or higher resource use. The calculated hazard ratio (HR) was adjusted for sex, age, income, comorbidities, DCSI score, staging of kidney disease, ADMs, and index year.

### Statistical analysis

We used 1:1 propensity score matching to reduce selection bias and optimize the variates of the case and control cohorts. The closest propensity score for the cases and controls was estimated. We used the nearest-neighbor algorithm to derive matched pairs, with values of standardized mean difference < 0.1, to indicate a significant difference between the cases and controls. Crude and multivariable-adjusted Cox proportional hazards models were used to compare the risk of outcomes of the cases and controls. We employed the Schoenfeld residuals test to assess if the model violates the assumption of proportional hazards. The results of the comparison are presented as HRs and 95% confidence intervals (CIs). We censored patients on the date of the respective outcomes, death, or withdrawal from the NHIRD or at the end of the follow-up on December 31, 2019, whichever came first. Kaplan–Meier analysis and log-rank tests were used to evaluate the cumulative incidence of DR in two cohorts during the follow-up. Furthermore, to assess the time effects of SGLT2is, we conducted a subgroup analysis using the pre-specified strata of the cumulative days, which were divided into three according to the population distribution (0–33%, 34–66%, and 67–100%). All statistical analyses were conducted using the SAS (version 9.5; SAS Institute, Cary, North Carolina, United States) platform, and two-tailed *P*-values of < 0.05 denoted statistical significance.

### Sensitivity analyses

Several sensitivity analyses were conducted to validate the robustness of our findings. For the first model (primary model), we assessed the effect of SGLT2is on DR progression using adjusted variables. For the second model, we performed an analysis based on an unmatched sample. For the third model, we selected only patients who underwent color fundoscopy or indirect ophthalmoscopy (procedure codes 23501C, 23502C, and 23702C) within 1 year before the index date. Therefore, the diagnostic codes for DR progression may be more reliable. The HR for DR progression with SGLT2is treatment was determined. Statistical significance was set at *P* < 0.05 for 2-side testing.

## Results

### Patient characteristics

**Figure 1:**
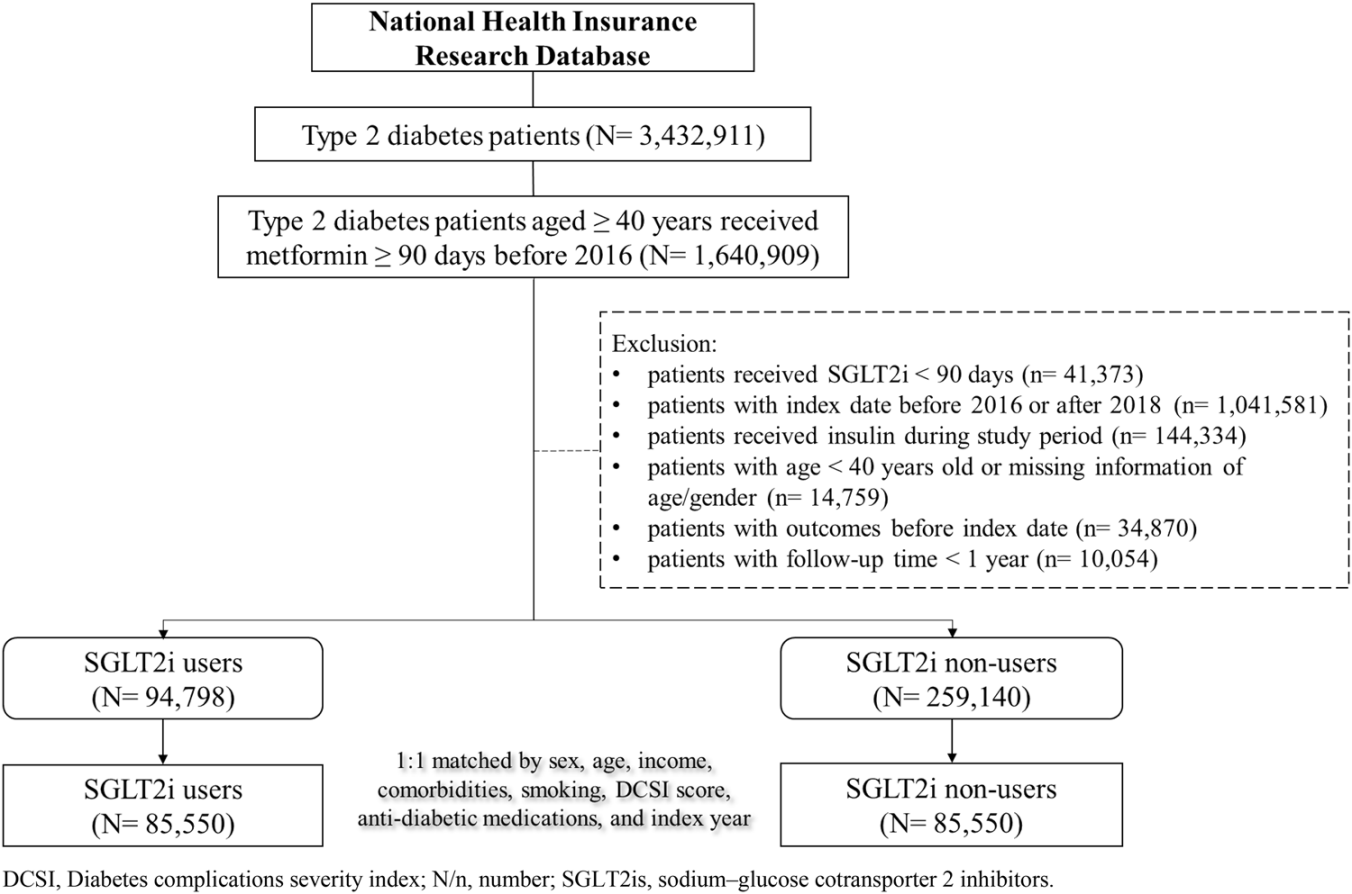
Study flow chart. *DCSI* diabetes complications severity index, *N/n* number, *SGLT2is* sodium–glucose cotransporter 2 inhibitors.

From January 1, 2016, to December 31, 2018, we identified 3,432,911 individuals with T2DM in the database. Of these, 1,640,909 received metformin for a duration longer than 90 days before 2016. After exclusion, 94,798 individuals received SGLT2is as add-on therapy, while 259,140 did not. Figure 1 shows the patient selection flowchart. After excluding ineligible patients, the 1:1 propensity score matching according to sex, age, income, comorbidities, smoking, DCSI score, ADMs, and index year were used to derive 85,550 pairs of patients treated with SGLT2is and those who were not. Table 1 shows the baseline characteristics of the study population. Females accounted for 44.34% of the participants in the SGLT2is cohort, and the mean age of the population was 60.74 ± 9.77 years. The overall mean follow-up duration of T2DM and DR were 7.78 ± 2.38 and 2.29 ± 0.77, respectively, for the patients receiving SGLT2is.

**Table 1:** Comparison of demographic characteristics and comorbidities between type 2 diabetes mellitus patients treated with or without sodium-glucose cotransporter 2 inhibitors.

Variables	Non-SGLT2is (N = 85,550)		SGLT2is (N = 85,550)		SMD
	N	%	N	%	
Sex					0.009
Female	38,323	44.80	37,936	44.34	
Male	47,227	55.20	47,614	55.66	
Age					
40–59	38,294	44.76	38,512	45.02	0.005
60–79	44,560	52.09	44,290	51.77	0.006
≥ 80	2696	3.15	2748	3.21	0.003
Mean (SD)	60.86	9.77	60.74	9.77	0.012
Income					
< 20,000	17,081	19.97	17,001	19.87	0.002
20,000–39,999	46,004	53.77	46,014	53.79	< 0.001
≥ 40,000	22,465	26.26	22,535	26.34	0.002
Comorbidity					
Hypertension	66,140	77.31	65,877	77.00	0.007
Dyslipidemia	74,263	86.81	73,779	86.24	0.017
CAD	25,923	30.30	26,241	30.67	0.008
CVA	10,970	12.82	11,268	13.17	< 0.001
Liver cirrhosis	988	1.15	1185	1.39	0.021
CKD	5157	6.03	5335	6.24	0.009
Obesity	3250	3.80	3387	3.96	0.008
Smoking	3511	4.10	3610	4.22	0.006
DCSI score					
0–1	18,383	21.49	17,853	20.87	0.015
2	17,408	20.35	17,413	20.35	< 0.001
≥ 3	49,759	58.16	50,284	58.78	0.012
Anti-diabetic medications					
Sus	66,094	53.77	64,995	52.88	0.030
TZDs	26,438	21.51	25,964	21.12	0.012
GLP1Ras	1537	1.25	1625	1.32	0.008
DPP4is	28,844	23.47	26,876	21.87	0.047
Types of anti-diabetic medications					
1	46,900	54.82	49,771	58.18	0.068
2	27,533	32.18	25,948	30.33	0.040
≥ 3	11,117	12.99	9831	11.49	0.046
Follow up period, mean (SD)					
T2DM	6.79	2.71	7.78	2.38	0.387
Diabetic retinopathy	2.32	0.84	2.29	0.77	0.045

1:1 propensity score matched by sex, age, income, comorbidities, smoking, DCSI score, staging of kidney disease, anti-diabetic medications, types of anti-diabetic medications, and follow-up period.

*CAD* coronary artery disease, *CKD* chronic kidney disease, *CVA* cerebrovascular accident, *DCSI* diabetes complications severity index, *DPP4is* dipeptidyl peptidase-4 inhibitors, *ESRD* end-stage renal disease, *GLP-1RAs* glucagon-like peptide 1 receptor agonists, *SD* standard deviation, *SMD* standardized mean difference, *SUs* sulfonylureas, *T2DM* type 2 diabetes mellitus, *TZDs* thiazolidinediones.

### Multivariate analyses

Table 2 shows the results of the multivariate Cox regression analysis of DR progression. A total of 819 (0.96%) patients who received SGLT2is add-on therapy develop DR progression during the follow-up with incidence rate 4.19 per 1000 person-years. Patients receiving SGLT2is add-on therapy showed a significantly decreased risk of DR (adjusted HR [aHR] 0.89, 95% CI 0.81–0.98, *P* = 0.0233) relative to the non-SGLT2is cohort. We also assessed the effects of other important clinical parameters on DR progression. Compared with individuals aged 40–59 years, those aged 60–79 years had a higher risk of DR after adjustment (aHR 1.13, 95% Cl 1.01–1.25; *P* = 0.0267). Patients with history of dyslipidemia, CAD, or liver cirrhosis had a significantly lower risk of DR. The risk of DR progression increased with increases in the DCSI scores. Individuals with DCSI score more than 3 had 1.72-fold hazards of DR progression than individuals with DCSI score less than 1 (95% Cl 1.48–2.00; *P* < 0.001). The use of SUs was associated with a remarkably increased risk of DR progression (aHR 1.61, 95% CI 1.39–1.86, *P* < 0.001).

**Table 2:** Hazard ratios and 95% confidence intervals of progression in diabetic retinopathy.

Variables	DR progression								
	n	PY	IR	cHR	95% CI	*P*	aHR^†^	95% CI	*P*
SGLT2is									
No	884	198,802.3	4.45	1.00	(Reference)	–	1.00	(Reference)	–
Yes	819	195,671.96	4.19	0.95	(0.86, 1.04)	0.2726	0.89	(0.81, 0.98)*	0.0233
Sex									
Female	780	176,808.44	4.41	1.00	(Reference)	–	1.00	(Reference)	–
Male	923	217,665.82	4.24	0.97	(0.88, 1.06)	0.5018	1.02	(0.92, 1.12)	0.7037
Age, year									
40–59	710	182,667.92	3.89	1.00	(Reference)	–	1.00	(Reference)	–
60–79	945	200,425.83	4.71	1.26	(1.14, 1.39)***	< 0.001	1.13	(1.01, 1.25)*	0.0267
≥ 80	48	11,380.50	4.22	1.19	(0.89, 1.60)	0.2370	1.02	(0.75, 1.38)	0.9043
Income									
< 20,000	361	77,952.36	4.63	1.00	(Reference)	–	1.00	(Reference)	–
20,000–39,999	898	211,403.79	4.25	0.91	(0.81, 1.03)	0.1500	0.92	(0.82, 1.04)	0.1978
≥ 40,000	444	105,118.11	4.22	0.90	(0.78, 1.03)	0.1337	0.95	(0.83, 1.10)	0.5064
Comorbidity									
Hypertension	1299	302,769.11	4.29	0.99	(0.88, 1.11)	0.8432	0.90	(0.81, 1.02)	0.0903
Dyslipidemia	1434	340,394.88	4.21	0.86	(0.75, 0.98)*	0.0217	0.83	(0.73, 0.95)**	0.0064
CAD	501	118,617.33	4.22	0.98	(0.89, 1.09)	0.7390	0.81	(0.72, 0.91)***	< 0.001
CVA	232	49,746.67	4.66	1.12	(0.97, 1.29)	0.1104	0.94	(0.81, 1.09)	0.4146
Liver cirrhosis	11	4727.96	2.33	0.56	(0.31, 1.01)	0.0550	0.52	(0.29, 0.94)*	0.0316
CKD	139	22,806.02	6.09	1.52	(1.27, 1.80)***	< 0.001	1.22	(1.01, 1.48)*	0.0378
Obesity	48	15,511.91	3.09	0.70	(0.53, 0.94)*	0.0160	0.80	(0.60, 1.06)	0.1237
Smoking	51	15,660.61	3.26	0.77	(0.59, 1.02)	0.0718	0.80	(0.61, 1.07)	0.1313
DCSI score									
0–1	265	86,006.14	3.08	1.00	(Reference)	–	1.00	(Reference)	–
2	291	81,830.76	3.56	1.16	(0.98, 1.37)	0.0770	1.16	(0.98, 1.38)	0.0780
≥ 3	1147	226,637.35	5.06	1.70	(1.48, 1.94)***	< 0.001	1.72	(1.48, 2.00)***	< 0.001
Anti-diabetic medications									
Sus	1482	308,609.43	4.80	1.75	(1.52, 2.01)***	< 0.001	1.61	(1.39, 1.86)***	< 0.001
TZDs	617	126,566.9	4.87	1.15	(1.04, 1.27)**	0.0055	1.07	(0.97, 1.19)	0.1792
GLP1Ras	41	8048.34	5.09	1.11	(0.82, 1.52)	0.5017	1.10	(0.81, 1.51)	0.5310
DPP4is	633	134,779.32	4.70	1.09	(0.99, 1.20)	0.0950	0.99	(0.90, 1.10)	0.8572

*CAD* coronary artery disease, *cHR* crude hazard ratio, *CKD* chronic kidney disease, *CVA* cerebrovascular accident, *DCSI* diabetes complications severity index, *DPP4is* dipeptidyl peptidase-4 inhibitors, *DR* diabetic retinopathy, *GLP-1RAs* glucagon-like peptide 1 receptor agonists, *IR* incidence rate, per 10,000 person-years, *PY* person-years, *SD* standard deviation, *SMD* standardized mean difference, *SUs* sulfonylureas, *TZDs* thiazolidinediones, *95% Cl* 95% confidence interval.

^†^Adjusted hazard ratio estimated by multivariable analysis including sex, age, income, comorbidities, and anti-diabetic medications.

*P < 0.05, **P < 0.01, ***P < 0.001.

Table 3 demonstrates the subgroup analysis for identifying the subpopulations that benefit most from the use of SGLT2is. Men who received SGLT2is add-on therapy had a significantly lower risk of DR progression (aHR 0.82, 95% CI 0.72–0.94; *P* = 0.0035). Individuals with higher incomes had a significantly lower risk of DR progression. SGLT2is users showed significantly lower HRs for developing CAD than non-SGLT2is users (aHR 0.79, 95% CI 0.66–0.95, *P* = 0.0112). Metformin and SGLT2is, in conjunction with other anti-diabetes medications, including SUs, TZDs, GLP-1RAs, and DPP4is, significantly reduced the risk of DR progression. Of note, combined use of metformin, SGLT2is, and GLP-1RAs can markedly decrease risk of DR progression (aHR 0.37, 95% CI 0.19–0.71, *P* = 0.0028).

**Table 3:** Subgroup analysis, stratified by sex, age, income, comorbidities, and other variables.

Variables	DR progression											
	Non-SGLT2is			SGLT2is			Univariate				Multivariate	
	N	PY	IR	N	PY	IR	cHR	95% CI	*P*	aHR^†^	95% CI	*P*
Sex												
Female	387	89,537.02	4.32	393	87,271.43	4.50	1.06	(0.92, 1.22)	0.4512	0.98	(0.85, 1.13)	0.7834
Male	497	109,265.3	4.55	426	108,400.53	3.93	0.87	(0.76, 0.99)*	0.0299	0.82	(0.72, 0.94)**	0.0035
Age, year												
40–59	368	91,838.7	4.01	342	90,829.22	3.77	0.94	(0.81, 1.09)	0.3869	0.94	(0.81, 1.09)	0.4137
60–79	489	101,299.9	4.83	456	99,125.93	4.60	0.97	(0.85, 1.10)	0.6106	0.97	(0.85, 1.10)	0.6016
≥ 80	27	5663.70	4.77	21	5716.80	3.67	0.79	(0.44, 1.40)	0.4160	0.78	(0.44, 1.40)	0.4088
Income												
< 20,000	176	39,439.50	4.46	185	38,512.86	4.80	1.09	(0.89, 1.34)	0.4119	1.02	(0.83, 1.26)	0.8371
20,000–39,999	462	106,605.8	4.33	436	104,797.95	4.16	0.97	(0.85, 1.11)	0.6448	0.89	(0.78, 1.02)	0.0896
≥ 40,000	246	52,756.96	4.66	198	52,361.15	3.78	0.81	(0.67, 0.98)*	0.0267	0.79	(0.65, 0.96)*	0.0182
Comorbidity												
Hypertension	671	152,840.4	4.39	628	149,928.72	4.19	0.96	(0.86, 1.07)	0.4855	0.91	(0.81, 1.02)	0.0947
Dyslipidemia	736	172,279.4	4.27	698	168,115.46	4.15	0.98	(0.89, 1.09)	0.7369	0.94	(0.84, 1.04)	0.2236
CAD	274	59,462.37	4.61	227	59,154.96	3.84	0.85	(0.71, 1.01)	0.0681	0.79	(0.66, 0.95)*	0.0112
CVA	114	24,686.09	4.62	118	25,060.58	4.71	1.05	(0.81, 1.36)	0.7329	0.95	(0.73, 1.23)	0.6747
Liver cirrhosis	5	2106.60	2.37	6	2621.36	2.29	0.95	(0.29, 3.12)	0.9366	0.99	(0.29, 3.40)	0.9914
CKD	61	11,295.22	5.40	78	11,510.80	6.78	1.24	(0.89, 1.74)	0.2033	1.17	(0.83, 1.65)	0.3568
Obesity	18	7469.23	2.41	30	8042.68	3.73	1.50	(0.83, 2.69)	0.1763	1.36	(0.75, 2.46)	0.3083
Smoking	33	7797.67	4.23	18	7862.94	2.29	0.55	(0.31, 0.99)*	0.0446	0.55	(0.30, 1.00)	0.0515
DCSI score												
0–1	141	44,167.38	3.19	124	41,838.77	2.96	0.93	(0.73, 1.19)	0.5630	0.89	(0.70, 1.15)	0.3800
2	152	41,250.98	3.68	139	40,579.78	3.43	0.93	(0.73, 1.17)	0.5103	0.85	(0.67, 1.08)	0.1913
≥ 3	591	113,383.9	5.21	556	113,253.41	4.91	0.95	(0.85, 1.07)	0.4058	0.90	(0.80, 1.02)	0.0879
Anti-diabetic medications												
Sus	771	156,557.9	4.92	711	152,051.49	4.68	0.96	(0.86, 1.06)	0.3808	0.89	(0.80, 0.99)*	0.0336
TZDs	346	64,206.48	5.39	271	62,360.42	4.35	0.81	(0.69, 0.95)**	0.0097	0.75	(0.64, 0.88)***	< 0.001
GLP1Ras	26	3653.38	7.12	15	4394.96	3.41	0.42	(0.22, 0.79)**	0.0072	0.37	(0.19, 0.71)**	0.0028
DPP4is	359	69,234.68	5.19	274	65,544.64	4.18	0.80	(0.68, 0.94)**	0.0060	0.77	(0.65, 0.90)**	0.0012

*CAD* coronary artery disease, *cHR* crude hazard ratio, *CKD* chronic kidney disease, *CVA* cerebrovascular accident, *DCSI* diabetes complications severity index, *DPP4is* dipeptidyl peptidase-4 inhibitors, *DR* diabetic retinopathy, *GLP-1RAs* glucagon-like peptide 1 receptor agonists, *IR* incidence rate, per 10,000 person-years, *PY* person-years, *SD* standard deviation, *SMD* standardized mean difference, *SUs* sulfonylureas, *TZDs* thiazolidinediones, *95% Cl* 95% confidence interval.

^†^Adjusted hazard ratio estimated by multivariable analysis including sex, age, income, comorbidities, and anti-diabetic medications.

*P < 0.05, **P < 0.01, ***P < 0.001.

### Cumulative incidence of DR progression

**Figure 2:**
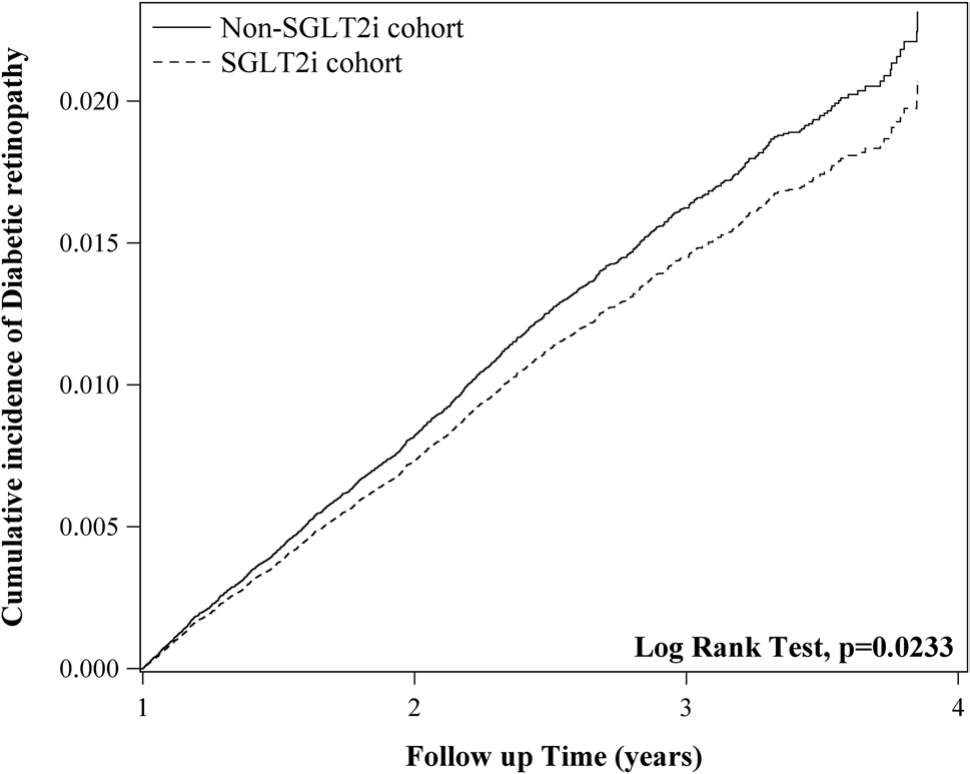
The cumulative incidence of diabetic retinopathy in SGLT2is and non-SGLT2is cohorts.

Figure 2 shows the Kaplan–Meier curve of the cumulative incidence rate of DR in patients who received and did not receive SGLT2is add-on therapy for metformin. The cumulative incidence of DR was significantly lower for the SGLT2is cohort than in the non-SGLT2is cohort in 4 years (log-rank *P* = 0.0233).

### Duration of use of SGLT2is

Table 4 shows the durations of SGLT2is add-on therapy. The adjusted risks of DR progression in patients receiving SGLT2is therapy for < 1 year and 1−2 years significantly increased (aHR 1.54 versus 1.86, both *P* < 0.001). With time, this changed, and the patients receiving add-on therapy for > 2 years had lower risks of DR (aHR 0.41, 95% CI 0.35 − 0.47; *P* < 0.001).

**Table 4:** The risks of progression of diabetic retinopathy in terms of different follow-up time.

Variables	DR progression									
	N	PY	IR	cHR	(95% CI)	*P*	aHR^†^	(95% CI)	*P*	*P* for trend
Controls	884	198,802.3	4.45	1.00	(Reference)	–	1.00	(Reference)	–	< 0.001
SGLT2is drug year										
< 1	196	31,742.96	6.17	1.51	(1.29, 1.76)***	< 0.001	1.54	(1.31, 1.81)***	< 0.001	
1–2	386	57,850.2	6.67	1.94	(1.72, 2.19)***	< 0.001	1.86	(1.64, 2.11)***	< 0.001	
> 2	237	106,078.8	2.23	0.44	(0.38, 0.51)***	< 0.001	0.41	(0.35, 0.47)***	< 0.001	

*cHR* crude hazard ratio, *DR* diabetic retinopathy, *IR* incidence rate, per 10,000 person-years, *PY* person-years, *95% Cl* 95% confidence interval.

^†^Adjusted hazard ratio estimated by multivariable analysis including sex, age, income, comorbidities, and anti-diabetic medications.

*P < 0.05, **P < 0.01, ***P < 0.001.

### Sensitivity analysis

Our primary findings were robust for the serial sensitivity analyses. For the first model (primary analysis), the aHR of SGLT2is effects on DR progression was 0.89 (95% CI 0.81−0.98, *P* = 0.0233). For the second model (Appendix Table 1), the aHR was 0.89 (95% CI 0.82−0.97, *P* = 0.0066) based on the unmatched cohort. We selected patients who underwent color fundoscopy or indirect ophthalmoscopy and were diagnosed with DR, for the reliability of their diagnoses, from the study population for further analysis of the risk of DR progression with SGLT2is treatment. For the third model (Appendix Table 2), the aHR was 0.81 (95% CI 0.70−0.94, *P* = 0.0051). Notably, individuals with a previous CAD diagnosis had a remarkably lower risk of DR progression in three models.

### Ethical statement

This retrospective cohort study was approved by institutional review board of China Medical University Hospital with number “CMUH110-REC3-133”. The informed consent was waived since the current study only aggregated counts and statistical summaries of de-identified information. Furthermore, protected health information was not collected, and no study-specific activities were performed in retrospective analyses.

## Discussion

This large-series cohort study demonstrated the following: (1) In participants with chronic kidney disease (CKD), the incidence rate of DR progression was higher than that of hypertension, dyslipidemia, CAD or cerebrovascular accident. (2) The risk of DR progression was observed to increase in individuals with higher DCSI scores and those who were using sulfonylureas. (3) Comorbid CKD participants had a higher risk of DR progression. (4) Individuals receiving SGLT2is add-on therapy with CAD had lower risk of DR progression than those without CAD. (5) The co-administration of metformin, SGLT2is, and other antidiabetic medications by participants was associated with a significant reduction in the risk of DR progression. (6) SGLT2is add-on therapy decreases the progression of DR in T2DM patients. (7) Patients who received SGLT2is add-on therapy for < 1 year had considerably elevated risks of DR, but the risk decreased markedly as the duration of therapy exceeded 2 years. In the current study, we chose a population older than 40 rather than 20 because, according to preliminary analysis, the proportion of people aged 20 to 39 was less than 5%. This was consistent with the findings of the Intelligent Research in Sight Registry, which found that 3.2% of retinal vascular occlusion patients were younger than 25 years old, while the majority of cases occurred between the ages of 65 and 85^15^.

The pathophysiology of DR involves the increased transfer of glucose from the circulation to the retina. High glucose concentrations inside the cells of the retina facilitate the synthesis of advanced glycation end products, which contribute to oxidative stress and consequently promote angiogenesis. Excess glucose concentrations in cells also facilitate various hazardous responses, such as overactivity of the hexosamine pathway and activation of the protein kinase C and polyol pathways. Induction of the hexosamine pathway results in increased glucose flux, which may lead to apoptosis of retinal neurons. Protein kinase C activation contributes to the loss of capillary pericytes, which is an early feature of DR^16^; hypoxia; and/or increased activity of the local renin-angiotensin system^17^. Activation of glial and neuronal abnormalities are mediated by the polyol pathway in experimental animals^18^. These signaling pathways interact to create a vicious loop that increases vascular permeability, retinal hypoxia, neural dysfunction, and neovascularization^19,20^. Finally, localized inflammation and the death of retinal and glial cells results in the worsening of DR. This loss of autoregulation results in increased retinal blood flow with an accompanying increase in shear stress on the retinal microvasculature.

The Taiwan Food and Drug Administration has approved canagliflozin, dapagliflozin, and empagliflozin for the treatment of T2DM since 2014, and these drugs have been covered by NHI reimbursement since May 1, 2016. They are typically used as second- or third-line treatments because other ADMs are available with complete safety profiles and lower costs^21^. SGLT2is are often used in combination for dual or triple therapies. Patients with major adverse cardiovascular events, defined as a composite of ischemic stroke, myocardial infarction, and sudden cardiac death and/or ventricular arrhythmia, can benefit from SGLT2is treatment^22,23^. SGLT2is also induce significant reductions of both systolic and diastolic blood pressures, which are among the most important risk factors of DR. These alterations are not dose-dependent and are more pronounced for systolic blood pressure^24^.

The effects of various ADMs on DR have been previously studied. Glucagon-like peptide (GLP-1), an incretin, is a gastrointestinal peptide that regulates glucose concentrations in the blood and is secreted in response to food consumption. It promotes insulin production and secretion only when food is orally ingested. GLP-1RAs can promote pancreatic islet β-cell insulin production, inhibit glucagon release, and limit glucose absorption by slowing gastric emptying and inhibiting glucagon secretion. Treatment with GLP-1RAs has not been associated with an increased risk of incident DR and may reduce the risk of DR 0.67-fold relative to insulin^25^. After treatment with exenatide, DR progressed from background retinopathy to bilateral proliferative DR (PDR) and DME^26^.

DPP4is are responsible for inactivating glucose-dependent insulinotropic polypeptide and GLP-1. The inhibition of this enzyme increases the availability of GLP-1 in the body, thereby boosting its activity. A retrospective study revealed that DPP4is may reduce the progression of DR^27^. However, the use of DPP4is for less than 12 months may be associated with early worsening of DR^28^.

TZD can activate genes that control lipid and glucose metabolism, enhance insulin sensitivity in the muscle and adipose tissue, and inhibit gluconeogenesis in the liver. Several population-based studies of TZD have been conducted. The use of rosiglitazone was related to a 59% relative risk reduction in the progression to PDR over 3 years and reduced rates of visual acuity loss^29^. Other studies have reported an association between TZD and an increased risk of DME^30–32^. Finally, some studies reported inconclusive results or found no connection between TZD and DME^33–35^.

SU binds to sulfonylurea receptors in pancreatic β-cells, resulting in the release of insulin. Gliclazide appears to work better than other SU medications in preventing the worsening of DR and progression to PDR^36^. Gliclazide may be more effective than glibenclamide for treating or preventing DR^37^. However, gliclazide does not affect diabetic microangiopathy^38^.

Metformin is the most used medication for the initial treatment of T2DM globally. It increases insulin sensitivity, decreases hepatic glucose production, and increases peripheral glucose absorption. Five-year metformin treatment is independently associated with a significant decrease in the incidence of non-proliferative DR and PDR in patients with T2DM for more than 15 years^39^. For individuals with DR who were receiving metformin monotherapy, the HR of DR progression was 0.74 for the DPP4i add-on group relative to the SU group^40^.

Early worsening is a term used to describe the worsening of DR at the commencement of diabetes treatment. This was initially observed in patients with type 1 diabetes mellitus who were rigorously treated with continuous subcutaneous insulin infusion as opposed to standard treatment with short- or intermediate-acting insulin^41^. The most significant predictors of DR early worsening were a higher HbA1c level at baseline and a decrease in this level throughout the first 6 months of treatment^42^. Regarding type 1 diabetes mellitus patients with non-to-moderate non-insulin-dependent hyperglycemia, early deterioration was detected in a considerably greater proportion of patients receiving intensive treatment than those receiving conventional treatment after 6 of 12 months (*P* < 0.001)^42^.

The evidence for early deterioration in patients with T2DM is limited because several large randomized controlled studies have only investigated the effect of anti-diabetic therapies on DR progression, as assessed by retinal change at the trial endpoint, and did not include early outcomes^43^. Early worsening has also been reported by retrospective case–control studies at several centers. A study reported that patients treated with intensive insulin had a higher reduction of HbA1c over 2 years than the control group (4.0% vs. 0.2%) and showed a 22.6% worsening of DR progression, while there was a negligible change from baseline in the controls^44^.

Evidence of the early deterioration of DR with non-insulin treatments is also increasing. In preliminary studies, the GLP-1RAs were associated with a temporary worsening of DR that resolved with continuing treatment^45^. Long-term studies have verified this transient condition, and adequate glycemic control has been shown to improve microvascular outcomes and DR. In a retrospective cohort analysis of individuals with T2DM, more than 6 months of treatment with exenatide resulted in the progression of DR in 29.7% of patients, whereas DR improved in 19.4% of patients. The proportion of patients with the progression of DR increased with reductions in HbA1c^46^. Prolonged treatment with exenatide resulted in improved (62%) or stable (18%) DR in 80% of patients after a mean of 439 days (1.2 years) from the phase 1 screening^45^.

Some hypotheses, including osmotic force, vascular endothelial growth factor (VEGF), synergy between hypoxia and VEGF, and potential effects of tight glycemic control, have been proposed to explain the early deterioration of DR^47,48^. Glucose is an osmotically active agent, and variations in blood glucose concentrations can affect osmotic pressure and, in turn, facilitate water retention and cause DME. The synergistic hypothesis postulates that early deterioration results from the synergistic effects of insulin and VEGF on retinal blood vessels, resulting in vascular proliferation^49^. Increased levels of VEGF were found in the retinas of diabetic rats treated with insulin^50^. In a hypoxic environment, tight glycemic control may result in VEGF overexpression. In in vitro experiments using human and bovine retinal cells, the absence of oxygen and glucose significantly upregulated VEGF production^51^.

Luseogliflozin has been observed to inhibit glucose absorption and increase VEGF type A (VEGF-A) expression in the kidneys of animals with the downregulation of glucose transporter 2 during ischemia/reperfusion and in glucose transporter 2-knock-down cells, relative to normal controls. Zhang et al.^52^ also found that luseogliflozin reduces glucose absorption and downstream VEGF-A expression. Treatment with SGLT2is normalized the concentration of VEGF-A in the podocytes of BTBR (black and tan, brachyuric) *ob/ob* mice^53^. In animal models of DR, empagliflozin decreased vascular leakage, as evidenced by the downregulation of VEGF in the retina, and changed the retinal genetic signature^54^. Tofogliflozin also improved retinal neurovascular coupling in T2DM mice by inhibiting VEGF expression and activating retinal glia^55^. In brief, SGLT2is may modulate neovascularization through the VEGF signaling pathway.

A possible mechanism of SGLT2is for the prevention of DR is that SGLT2is is the preservation of the delicate nutrition metabolism of the eye^56^. According to a study involving relatively few patients, SGLT2is help moderate hyperglycemia, hypertension, and hyperlipidemia and may prevent DR progression^57^. They also have protective effects on the fundus capillaries^58^, blood-retinal barrier^9,59^, and optic nerve^60^.

HbA1c, blood pressure, and serum total cholesterol may account for only 15% of the risk of DR. This suggests that genetic, environmental, or other factors may play key roles in the development of DR. As an example, uric acid can stimulate the inflammation of the retina and increase the activation of the Notch signaling system based on the observations of a high-glucose in vitro model^61^. Patients with DR have considerably higher uric acid concentrations than diabetic controls and healthy participants^62^. Nearly all SGLT2is have been found to increase uric acid excretion in T2DM patients^63,64^.

### Strengths and limitations

The major strength of our study is that it used real-world data to demonstrate that SGLT2is add-on therapy can delay the progression of DR. Taiwan’s NHI program is mandatory and covers approximately all people of this country. This nationwide cohort study recruited patients from the NHIRD, which may reduce selection bias. Nevertheless, this study has some limitations. First, DR was defined using diagnostic codes in the database; misclassification due to coding errors or misdiagnosis may be an issue. The accuracy of diagnoses cannot be confirmed using medical records. However, the Taiwan NHI has established a system that will allow interviewing patients and reviewing medical charts to verify the validity of the diagnosis and quality of care. We identified the study population with at least two outpatient visits or one hospitalization record to prevent miscoding. Furthermore, sensitivity analyses revealed consistent findings. Second, DR, which may be asymptomatic for several years, can be difficult to detect using incident cases in a medical record database setting. Third, the NHIRD database lacks some relevant clinical and laboratory information, such as fundus photographic recordings, HbA1c and C-reactive protein. An increase in C-reactive protein is associated with increased risks of clinically severe macular edema and DR^65,66^. Fourth, although adjusted for various confounding factors, more studies in clinical practice are needed to confirm the protective effects of SGLT2is. A cohort study is usually associated with bias due to uncovered and unobserved confounding factors, and a randomized controlled trial is warranted to verify our results. Although randomized controlled studies may verify or refute the present findings, they are impractical and time-consuming. Therefore, this high-quality observational study provides the strongest evidence on this topic. Lastly, the findings of the present study may only be related to the Taiwanese population; thus, other similar studies should be performed in different countries to check if the association holds.

## Conclusion

In summary, this population-based cohort study showed that anti-diabetic treatment with an SGLT2is add-on was associated with a significantly higher risk of DR progression in the short term but a remarkably lower risk of DR progression in the long term. For DR patients, the continued SGLT2is add-on therapy may yield benefits beyond glycemic control. The eyes of patients with an elevated ocular risk should be evaluated before commencing aggressive therapies to achieve glycemic control. Finally, long-term treatment with an SGTL2i without interruption may effectively prevent DR progression.

### Supplementary Information

Supplementary Tables.

## Data Availability

Data are available upon reasonable request and would be provided by the corresponding author.

## Abbreviations

ACEi: Angiotensin-converting enzyme inhibitor
ADM: Anti-diabetic medication
ARB: Angiotensin receptor blocker
CAD: Coronary artery disease
DPP4: Dipeptidyl peptidase-4
DPP4i: Dipeptidyl peptidase-4 inhibitor
DR: Diabetic retinopathy
DM: Diabetes mellitus
DME: Diabetic macular edema
GLP-1: Glucagon-like peptide 1
GLP-1RA: Glucagon-like peptide 1 receptor agonist
PDR: Progressive diabetic retinopathy
SGLT2i: Sodium-glucose cotransporter 2 inhibitor
SU: Sulfonylurea
TZD: Thiazolidinedione
T2DM: Type 2 diabetes mellitus
95% CI: 95% Confidence interval
