# Supplementary material for: Sodium-glucose co-transporter 2 inhibitor add-on therapy for metformin delays diabetic retinopathy progression in diabetes patients: a population-based cohort study
Source: Sci Rep. 2023 Oct 10;13:17049. doi: 10.1038/s41598-023-43893-2 (PMC10564914; doi:10.1038/s41598-023-43893-2)
Supplement: Supplementary file 1 — Supplementary Tables. [file 41598_2023_43893_MOESM1_ESM.docx]

**Appendix Table 1. Sensitivity analysis based on unmatched cohorts**

|  | DR progression | | | |  |  |  |  |  | |  |
| --- | --- | --- | --- | --- | --- | --- | --- | --- | --- | --- | --- |
| Variables | n | PY | IR | cHR | 95% CI | *P* | aHR^†^ | 95% CI | | *P* | |
| SGLT2is |  |  |  |  |  |  |  |  | |  | |
| No | 2627 | 634392.97 | 4.14 | 1.00 | (reference) | - | 1.00 | (reference) | | - | |
| Yes | 905 | 215577.2 | 4.20 | 1.07 | (0.99, 1.15) | 0.1005 | 0.89 | (0.82, 0.97)** | | 0.0066 | |
| Sex |  |  |  |  |  |  |  |  | |  | |
| Female | 1650 | 401847.47 | 4.11 | 1.00 | (reference) | - | 1.00 | (reference) | | - | |
| Male | 1882 | 448122.71 | 4.20 | 1.03 | (0.96, 1.10) | 0.4189 | 1.05 | (0.98, 1.12) | | 0.1699 | |
| Age, year |  |  |  |  |  |  |  |  | |  | |
| 40–59 | 1191 | 322340.82 | 3.69 | 1.00 | (reference) | - | 1.00 | (reference) | | - | |
| 60–79 | 2055 | 460382.48 | 4.46 | 1.23 | (1.15, 1.32)*** | <0.001 | 1.16 | (1.07, 1.25)*** | | <0.001 | |
| ≥80 | 286 | 67246.88 | 4.25 | 1.21 | (1.07, 1.38)** | 0.0032 | 1.13 | (0.99, 1.31) | | 0.0793 | |
| Income |  |  |  |  |  |  |  |  | |  | |
| <20000 | 806 | 190119.75 | 4.24 | 1.00 | (reference) | - | 1.00 | (reference) | | - | |
| 20000–39999 | 1914 | 456658.64 | 4.19 | 0.99 | (0.91, 1.07) | 0.7715 | 0.99 | (0.91, 1.07) | | 0.7312 | |
| ≥40000 | 812 | 203191.79 | 4.00 | 0.94 | (0.85, 1.04) | 0.2150 | 0.97 | (0.88, 1.07) | | 0.5174 | |
| Comorbidity |  |  |  |  |  |  |  |  | |  | |
| Hypertension | 2716 | 647106.5 | 4.20 | 1.06 | (0.98, 1.15) | 0.1316 | 0.97 | (0.90, 1.06) | | 0.5369 | |
| Dyslipidemia | 2840 | 702216.25 | 4.04 | 0.88 | (0.81, 0.96)** | 0.0024 | 0.85 | (0.78, 0.92)*** | | <0.001 | |
| CAD | 981 | 240286.37 | 4.08 | 0.99 | (0.92, 1.07) | 0.8668 | 0.84 | (0.78, 0.91)*** | | <0.001 | |
| CVA | 576 | 134771.31 | 4.27 | 1.05 | (0.96, 1.15) | 0.2421 | 0.91 | (0.83, 1.01) | | 0.0645 | |
| Liver cirrhosis | 53 | 13676.29 | 3.88 | 0.97 | (0.74, 1.27) | 0.7982 | 0.91 | (0.69, 1.19) | | 0.4916 | |
| CKD | 325 | 60519.92 | 5.37 | 1.38 | (1.23, 1.55)*** | <0.001 | 1.12 | (0.98, 1.27) | | 0.0984 | |
| Obesity | 75 | 26495.59 | 2.83 | 0.68 | (0.54, 0.85)*** | <0.001 | 0.73 | (0.58, 0.92)** | | 0.0067 | |
| Smoking | 109 | 30414.17 | 3.58 | 0.89 | (0.73, 1.07) | 0.2195 | 0.90 | (0.74, 1.09) | | 0.2894 | |
| DCSI score |  |  |  |  |  |  |  |  | |  | |
| 0–1 | 655 | 203482.40 | 3.22 | 1.00 | (reference) | - | 1.00 | (reference) | | - | |
| 2 | 633 | 170517.75 | 3.71 | 1.17 | (1.05, 1.31)** | 0.0044 | 1.16 | (1.04, 1.30)** | | 0.0077 | |
| ≥3 | 2244 | 475970.03 | 4.71 | 1.53 | (1.40, 1.67)*** | <0.001 | 1.52 | (1.38, 1.69)*** | | <0.001 | |
| Anti-diabetic medications |  |  |  |  |  |  |  |  | |  | |
| SUs | 2477 | 497100.75 | 4.98 | 1.63 | (1.52, 1.76)*** | <0.001 | 1.57 | (1.45, 1.69)*** | | <0.001 | |
| TZDs | 855 | 173325.11 | 4.93 | 1.23 | (1.14, 1.33)*** | <0.001 | 1.08 | (1.00, 1.17) | | 0.0577 | |
| GLP1RAs | 61 | 12421.59 | 4.91 | 1.13 | (0.88, 1.46) | 0.3410 | 1.05 | (0.81, 1.36) | | 0.7017 | |
| DPP4is | 1502 | 332673.08 | 4.51 | 1.12 | (1.04, 1.19)** | 0.0012 | 0.98 | (0.91, 1.05) | | 0.5389 | |

CAD, coronary artery disease; cHR, crude hazard ratio; CKD, chronic kidney disease; CVA, cerebrovascular accident; DCSI, Diabetes complications severity index; DPP4is, dipeptidyl peptidase-4 inhibitors; DR, diabetic retinopathy; GLP-1RAs, glucagon-like peptide 1 receptor agonists; IR, incidence rate, per 10,000 person-years; PY, person-years; SD, standard deviation; SMD, standardized mean difference; SUs, sulfonylureas; TZDs, thiazolidinediones; 95% Cl, 95% confidence interval.

^†^ Adjusted hazard ratio estimated by multivariable analysis including sex, age, income, comorbidities, DCSI score, smoking, anti-diabetic medications, and index year.

**P*<0.05, ***P*<0.01, ****P*<0.001.

**Appendix Table 2. Sensitivity analysis based on the individuals with color fundoscopy or indirect ophthalmoscopy to confirm state of diabetic retinopathy one year prior to the outcome date**

|  | DR progression | | | |  | |  | |  | |  | |  |  |
| --- | --- | --- | --- | --- | --- | --- | --- | --- | --- | --- | --- | --- | --- | --- |
| Variables | n | PY | IR | cHR | | 95% CI | | *P* | | aHR^†^ | | 95% CI | | *P* |
| SGLT2is |  |  |  |  | |  | |  | |  | |  | |  |
| No | 430 | 81241.47 | 5.29 | 1.00 | | (reference) | | - | | 1.00 | | (reference) | | - |
| Yes | 355 | 80204.06 | 4.43 | 0.84 | | (0.73, 0.97)* | | 0.0162 | | 0.81 | | (0.70, 0.94)** | | 0.0051 |
| Sex |  |  |  |  | |  | |  | |  | |  | |  |
| Female | 389 | 77615.01 | 5.01 | 1.00 | | (reference) | | - | | 1.00 | | (reference) | | - |
| Male | 396 | 83830.52 | 4.72 | 0.95 | | (0.83, 1.09) | | 0.4673 | | 1.01 | | (0.87, 1.16) | | 0.9438 |
| Age, year |  |  |  |  | |  | |  | |  | |  | |  |
| 40–59 | 272 | 66756.25 | 4.07 | 1.00 | | (reference) | | - | | 1.00 | | (reference) | | - |
| 60–79 | 491 | 89934.20 | 5.46 | 1.39 | | (1.20, 1.61)*** | | <0.001 | | 1.27 | | (1.08, 1.48)** | | 0.0039 |
| ≥80 | 22 | 4755.07 | 4.63 | 1.25 | | (0.81, 1.93) | | 0.3143 | | 1.11 | | (0.71, 1.74) | | 0.6502 |
| Income |  |  |  |  | |  | |  | |  | |  | |  |
| <20000 | 149 | 31646.49 | 4.71 | 1.00 | | (reference) | | - | | 1.00 | | (reference) | | - |
| 20000–39999 | 445 | 87808.36 | 5.07 | 1.08 | | (0.89, 1.29) | | 0.4409 | | 1.11 | | (0.92, 1.34) | | 0.2563 |
| ≥40000 | 191 | 41990.68 | 4.55 | 0.96 | | (0.77, 1.19) | | 0.7000 | | 1.04 | | (0.84, 1.3) | | 0.6986 |
| Comorbidity |  |  |  |  | |  | |  | |  | |  | |  |
| Hypertension | 594 | 120966.82 | 4.91 | 1.05 | | (0.90, 1.24) | | 0.5225 | | 0.96 | | (0.81, 1.14) | | 0.6268 |
| Dyslipidemia | 661 | 139637.70 | 4.73 | 0.85 | | (0.70, 1.03) | | 0.0880 | | 0.83 | | (0.69, 1.01) | | 0.0643 |
| CAD | 212 | 45457.93 | 4.66 | 0.97 | | (0.83, 1.13) | | 0.6704 | | 0.80 | | (0.67, 0.94)** | | 0.0086 |
| CVA | 111 | 20750.39 | 5.35 | 1.14 | | (0.93, 1.40) | | 0.1923 | | 0.98 | | (0.79, 1.2) | | 0.8274 |
| Liver cirrhosis | 9 | 2186.97 | 4.12 | 0.88 | | (0.45, 1.69) | | 0.6908 | | 0.84 | | (0.43, 1.62) | | 0.5991 |
| CKD | 62 | 10726.94 | 5.78 | 1.27 | | (0.98, 1.64) | | 0.0732 | | 1.14 | | (0.86, 1.51) | | 0.3723 |
| Obesity | 21 | 6492.29 | 3.23 | 0.65 | | (0.42, 1.00)* | | 0.0495 | | 0.75 | | (0.48, 1.16) | | 0.1927 |
| Smoking | 23 | 6103.89 | 3.77 | 0.80 | | (0.53, 1.21) | | 0.2842 | | 0.85 | | (0.56, 1.3) | | 0.4588 |
| DCSI score |  |  |  |  | |  | |  | |  | |  | |  |
| 0–1 | 88 | 27757.15 | 3.17 | 1.00 | | (reference) | | - | | 1.00 | | (reference) | | - |
| 2 | 143 | 32996.53 | 4.33 | 1.38 | | (1.06, 1.80)* | | 0.0177 | | 1.38 | | (1.05, 1.80)* | | 0.0192 |
| ≥3 | 554 | 100691.85 | 5.50 | 1.80 | | (1.44, 2.25)*** | | <0.001 | | 1.83 | | (1.43, 2.34)*** | | <0.001 |
| Anti-diabetic medications |  |  |  |  | |  | |  | |  | |  | |  |
| SUs | 657 | 122316.65 | 5.37 | 1.54 | | (1.28, 1.87)*** | | <0.001 | | 1.41 | | (1.16, 1.71)*** | | <0.001 |
| TZDs | 263 | 48739.27 | 5.40 | 1.12 | | (0.97, 1.30) | | 0.1201 | | 1.05 | | (0.91, 1.23) | | 0.4945 |
| GLP1RAs | 23 | 4424.81 | 5.20 | 1.01 | | (0.67, 1.54) | | 0.9446 | | 1.08 | | (0.71, 1.65) | | 0.7071 |
| DPP4is | 303 | 60450.80 | 5.01 | 1.00 | | (0.87, 1.16) | | 0.9769 | | 0.94 | | (0.81, 1.09) | | 0.3999 |

CAD, coronary artery disease; cHR, crude hazard ratio; CKD, chronic kidney disease; CVA, cerebrovascular accident; DCSI, Diabetes complications severity index; DPP4is, dipeptidyl peptidase-4 inhibitors; DR, diabetic retinopathy; GLP-1RAs, glucagon-like peptide 1 receptor agonists; IR, incidence rate, per 10,000 person-years; PY, person-years; SD, standard deviation; SMD, standardized mean difference; SUs, sulfonylureas; TZDs, thiazolidinediones; 95% Cl, 95% confidence interval.

^†^ Adjusted hazard ratio estimated by multivariable analysis including sex, age, income, comorbidities, DCSI score, smoking, anti-diabetic medications, and index year.

**P*<0.05, ***P*<0.01, ****P*<0.001.
